# Remarks on the Surgeons' Bill

**Published:** 1817-03

**Authors:** 


*For the London Medical and Physical Journal.*

### *Remarks on the Surgeons' Bill*; by A FRIEND TO RATIONAL REFORM.

ALLOW me, through the medium of your impartially-conducted Journal, to ask, from some one of your numerous readers, information on some parts of the Surgeons' Bill; and on some recent regulations connected with the public Lectures at the Royal College.

1st. As the Royal College say to Parliament, that, "from ignorant and incapable persons practising, the lives of many are destroyed," will the competence of future practitioners in *midwifery* be provided for to the satisfaction of the said College, by their becoming tributary to them, who are precluded from practically knowing any thing of the matter,— their their own bye-laws enforcing, "that no person who practises midwifery shall be elected a member of the Court of Assistants or Examiners;" and again, "that, if any person, being of the Court of Assistants, shall thereafter, or while he shall continue a member of such Court, practise as a man-midwife, he shall be liable to such fine, to the use of the College, as the Court of Assistants shall adjudge, not exceeding the sum of *fifty pounds* for each and every week in, or during, which he shall so continue to practise."* Do then the learned professors, by receiving fees for examinations, suppose that they can secure to the public a supply of competent men-midwives.

2dly. Do they consider the loss of lives they lament to have arisen from a want of competence in their own diplomatists, rather than from the many irregular and self-created surgeons who have intruded into the profession? and, in proposing to enforce *a year's* attendance in town for every future practitioner, do they provide for a continuance of the various lectures and dissections during the dog-days? Otherwise do they not fear that a summer spent *idly* in a metropolis abounding with fascinating allurements, may rather deteriorate than improve the intellectual acquirements and moral habits of the students? and, further, do they consider the present times as peculiarly adapted for imposing new burthens upon any part of his Majesty's subjects?

3dly. As the sums paid to the Royal College for diplomas, by members believed to be alive when the late list was published, amount to between *sixty* and *seventy thousand pounds*—as they must also have received, and be in the habit of receiving, numerous fees for examining surgeons and assistant-surgeons for the army, navy, East-India service, &c. and upon binding apprentices—and, as they now propose, by the Bill in question, to make every future practitioner of midwifery as well as surgery throughout the kingdom tributary to themselves, or to a Scotch or Irish College,—do they intend to apportion some part of this new income to the support of the College, and to desist from levying any further contributions upon their members, as being unnecessary, and, consequently, unjust?

4thly. As a 'College' is understood to mean 'a society of men set apart for learning,' and as the hall of such society is the joint property of the members or constituents of such College, who are bound, in the present case, "to pay annually the sum of twenty shillings towards defraying the

---

* **Bye-Laws, &c. of the Royal College of Surgeons, page 27.**

necessary necessary charges and expenses of the College,"* is it *lawful*, and, if lawful, is it *courteous*, for the Court of Assistants to refuse admittance by the usual and common entrance, to their members and co-proprietors, on all public occasions? Do the Royal College of Physicians, or the Society of Apothecaries, or any corporate body in the kingdom, when they

"——— on the beadle call,
To summon all the members to the hall,"†

oblige them to enter by dark ways and passages? and do not the very fundamental laws of the said Royal College of Surgeons say, "that no member shall lose, or be in danger of losing, any of the rights and privileges to which, as a member of the College, he is entitled?"

### COLLECTANEA MEDICA,

* Bye-Laws of the Royal College of Surgeons, page 24.
† Garth.

